# Superlensing enables radio communication and imaging underwater

**DOI:** 10.1038/s41598-023-45663-6

**Published:** 2023-10-26

**Authors:** Igor I. Smolyaninov, Quirino Balzano, Mark Barry, Dendy Young

**Affiliations:** Saltenna LLC, 1751 Pinnacle Drive #600, McLean, VA 22102 USA

**Keywords:** Ocean sciences, Physics

## Abstract

Wireless radio communications provide a backbone to our technological civilization. However, radio communications are widely believed to be impossible in many situations where radios are surrounded by conductive media, such as underwater or underground, thus making ocean exploration difficult and creating well-known mine safety problems. In addition, since most imaging techniques rely on electromagnetic waves, the difficulty of electromagnetic wave propagation through biological tissues, which are mostly made of water, also severely limits bioimaging. Here we show that contrary to common beliefs, radio signals may be efficiently propagated through water over useful distances. Both radio communication and radio imaging through water may be enabled by superlensing of surface electromagnetic waves propagating along the water surface. We have demonstrated underwater radio communication over distances of several hundred skin depth in the MHz frequency range, which would require sensitivity below 10^−100^ W in a conventional radio communication channel. We also demonstrated subwavelength super-resolution radio imaging in the GHz range by using water surface as a superlens. Our results indicate new ways to perform bioimaging, as well as marine life safe techniques of wireless radio communication and imaging underwater, which are essential for ocean and seafloor exploration. We also anticipate that the developed techniques will provide invaluable means of studying the extraterrestrial water worlds, such as potentially inhabitable Jovian moons.

Based on their electromagnetic properties in a certain frequency range, all macroscopic media may be separated into two broad classes of transparent and non-transparent media. Transparent media support electromagnetic waves propagation over long distances, while non-transparent (often called conductive) media do not. For example, water is relatively transparent only in the visible frequency range, while radio signals cannot be transmitted over long distance through bulk water. It is generally assumed that all electromagnetic waves quickly decay inside the non-transparent conductive media over short length scales of the order of the so-called skin depth δ. If a source of radio waves is placed deep inside a large body of water, the electric field at a distance *r* away from the source behaves as

1 $$E_{volume} \, \sim \,\frac{1}{r}e^{{ - \frac{r}{\delta }}} .$$

The radio signal strength *P* ~ *E*^*2*^ decays roughly by a factor of 10 (or − 10 dB) over each successive skin depth separation from the source, as illustrated by the green circle in Fig. 1a. The skin depth of radio waves in water is very short^1^:

2 $$\delta \, = \,{\sqrt {\frac{1}{{\pi \mu_{0} \sigma \nu }}} \approx \frac{{271m \cdot Hz^{1/2} }}{\sqrt \nu },}$$

where μ_0_ is the magnetic permeability of vacuum, σ is water conductivity, υ is the radio frequency, and the numerical value of δ is given for seawater conductivity σ = 3.5S/m. For example, at υ = 1 MHz the skin depth in seawater equals δ = 0.27 m. Therefore, transmission of radio waves through water is widely believed to be impossible even at very large transmit power. Indeed, if the volume transmission channel is used for radio communication underwater, a change in transmit power from *P* = 1W to P = 1 MW only gains an additional 6δ in communication distance. Since rocks and soils typically contain substantial amounts of water, radio communication underground is also very limited, thus creating well-publicized difficulties in search and rescue underground operations. The same limitations also make radio imaging prohibitively difficult underwater and through water-containing media, such as biological tissues.

**Figure 1:**
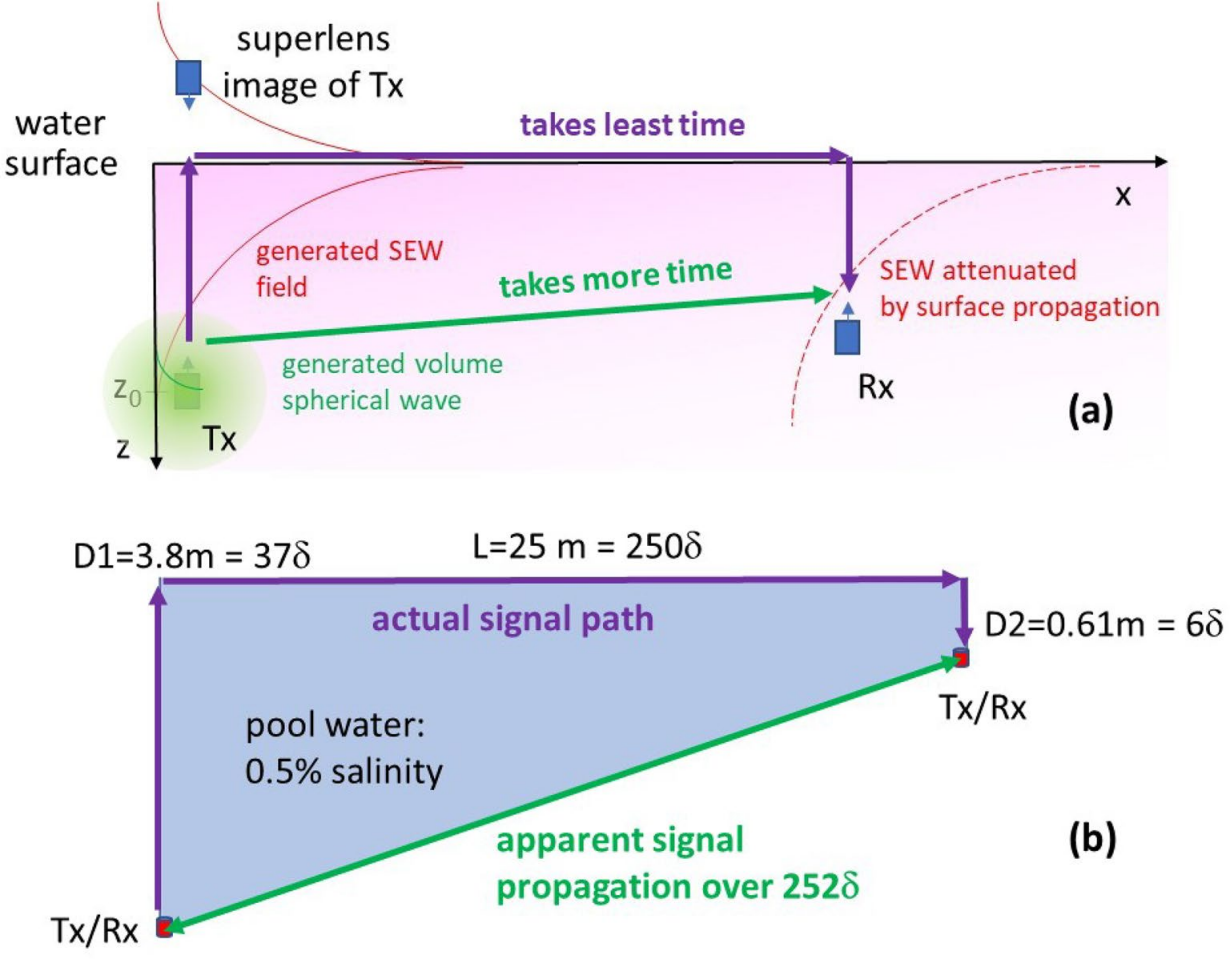
(**a**) Propagation of radio signals underwater from a transmitter Tx to a receiver Rx via the surface electromagnetic wave (SEW) channel. While the spherical volume wave shown by the green circle quickly decays on the scale of a few skin depth δ, the fraction of radio signal which is coupled to the SEW mode propagates much farther along the water surface, and it is received by the Rx. Note that a superlens image of Tx appears above the water surface. It is interesting to note that signal propagation via the surface wave channel is also favored by the Fermat’s principle. The signal path along the water surface takes considerably shorter time compared to the straight-line path underwater, which is much slower. (**b**) Schematic diagram of one of the radio communication experiments in a swimming pool (water salinity 0.5%, δ ≈ 0.1 m) performed at 50 MHz at 5 W transmit power. The distance and depths of the radios are expressed in terms of the number of skin depth δ in pool water. Conventional radio communication over the apparent path of 252δ through bulk water would require radio receiver sensitivity of ~10^-100^ W.

The goal of this paper is to demonstrate that the difficulty of radio communication and imaging underwater may be alleviated by making use of an alternative surface communication channel. In fact, as illustrated in Fig. 1a, the surface channel is strongly favored by Fermat’s least time principle^2^. Since water in the RF range has very large refractive index, the signal path along the water surface takes considerably shorter time compared to the straight-line path between radios underwater, which is much slower. Very recently it was demonstrated that such surface communication channels may indeed exist at the seawater-air and seawater-sea floor interfaces^3^. If an underwater radio transmitter is capable of coupling to the surface electromagnetic wave (SEW) propagating along the seawater-air interface, the electric field of such SEW signal far from the source will behave as

3 $$E_{SEW} \, \sim \,\frac{1}{{\rho^{1/2} }}e^{{ - \frac{z}{{\delta^{\prime } }}}} e^{{ - \frac{\rho }{\delta^*}}} ,$$

where *z* is the depth underwater, δ*’* is the SEW penetration depth into water, ρ is the radial distance from the source, and δ* is the SEW propagation length (note that in general δ*’* > δ and it is expected that δ* >  > δ, see Ref.^3^ and the “Methods” section). If one follows the signal path favored by Fermat’s principle, Eq. (3) indicates that the electric field of the radio signal increases exponentially towards the water surface, is lightly attenuated while propagating along the surface, and decreases exponentially towards the receiver location. The net result of this process is that a radio receiver (located at approximately the same depth as transmitter underwater) recovers almost the same radio signal, which is only weakly attenuated by a surface propagation factor of $e^{{ - \frac{\rho }{\delta *}}}$, as compared to the complete suppression of the conventional radio signal by the bulk propagation factor of $e^{{ - \frac{r}{\delta }}}$ along the straight-line path (see Eq. (1)). As will be discussed later in more detail, a superlens image^4,5^ of the radio transmitter must also appear above the water surface, as illustrated in Fig. 1a, thus enabling direct radio communication through the water surface. Since these kinds of exponential attenuation and recovery of the electromagnetic fields are typically involved in the superlensing effects^4–6^, we may say that superlensing effects enable radio communication and imaging underwater.

Our underwater radio communication experiments performed using SEW antennas in the MHz frequency range indeed demonstrate efficient radio communication over distances of many hundreds of skin depth—see for example the experimental geometry of a typical 50 MHz experiment depicted in Fig. 1b. In these experiments two Yaesu VX-8 radios operated at 50 MHz at 5 W output power were connected to their respective SEW antennas (as described in detail in Ref.^7^) and used for voice communication, while both divers and all the components of their radio systems were completely submerged underwater. Similar underwater experiments were also performed in freshwater (see Fig. 2a) and seawater. Quantitative analysis of these experiments provides strong evidence of the SEW transmission mechanism underwater. When plotted as distance *L* versus the sum of diver depths *D*_*1*_ and *D*_*2*_ underwater (see Fig.2b and c), the experimental datapoints exhibit a well-defined linear dependence. For example, radio communication in a freshwater lake was successful for all datapoints located inside the grey triangle in the plot in Fig. 2b. The slope of this dependence clearly points at the SEW communication mechanism. Indeed, in the absence of external RF noise, radio communication underwater is limited by the constant radio receiver sensitivity, so that

4 $$e^{{ - \frac{{D_{1} }}{\delta ^{\prime}}}} e^{{ - \frac{{D_{2} }}{\delta ^{\prime}}}} e^{{ - \frac{L}{\delta^*}}} \approx const$$

**Figure 2:**
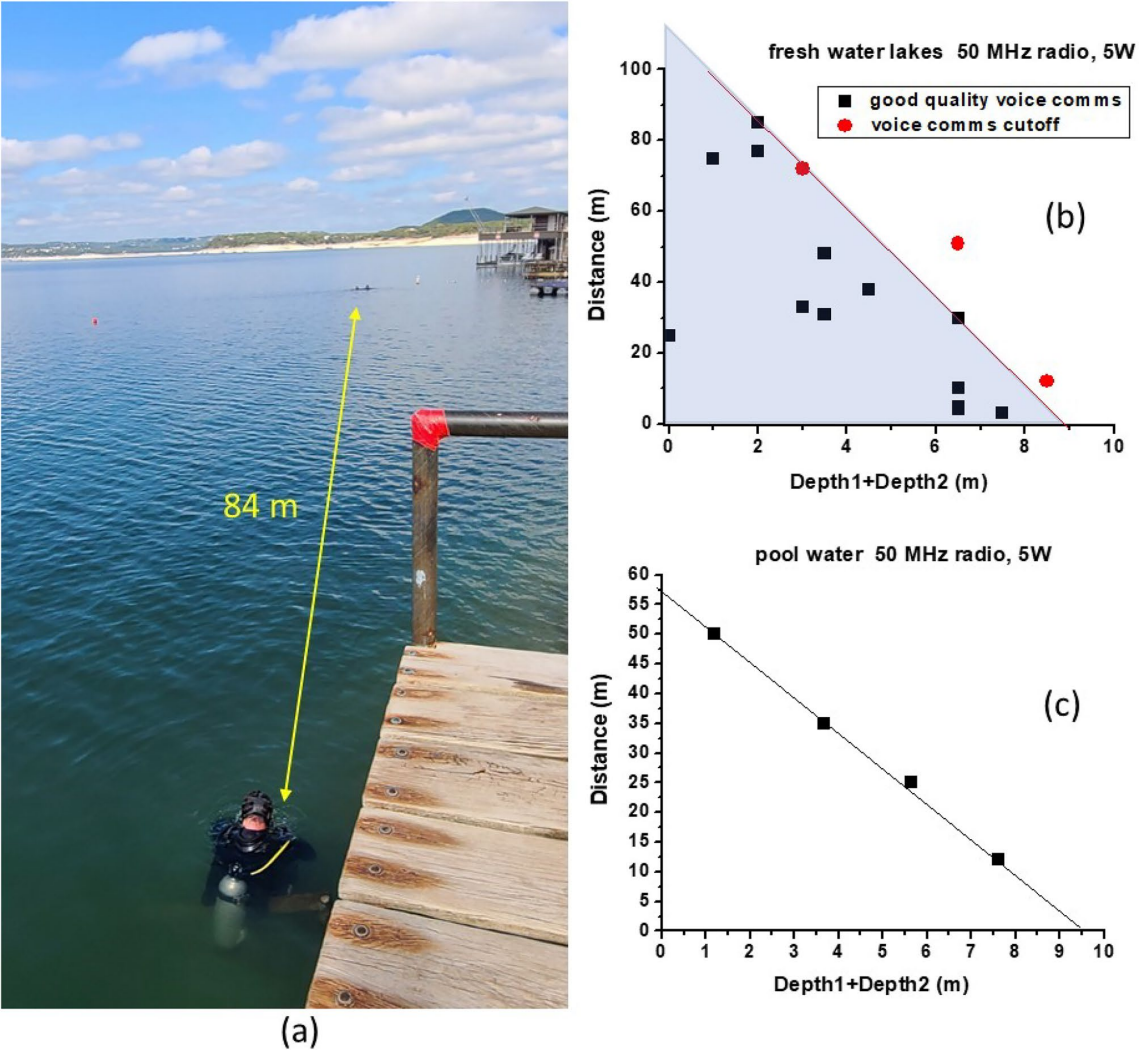
(**a**) Photo of an underwater radio communication experiment performed at 50 MHz, and results of such experiments performed in (**b**) fresh water (δ = 0.4m) and (**c**) pool water (δ = 0.1m) plotted as communication distance vs. the sum of depths of the two radios underwater. In both cases radio communication was achieved over distances of many hundreds of skin depth.

If the non-exponential factors in Eq. (3) are neglected. The logarithm of Eq. (4) gives rise to the linear dependence observed in the experiment:

5 $$L \approx C - \frac{\delta^*}{{\delta ^{\prime}}}\left( {D_{1} + D_{2} } \right)$$

Based on the experimental datapoints in Fig. 2b, it appears that δ*/δ’ > 12, which confirms the SEW character of the radio communication links underwater, and which clearly explains the observed combinations of radio communication depths and distances far beyond the conventional limit set by Eq. (1). For example, radio communication over an apparent path of 252δ through bulk water depicted in Fig. 1b would require radio receiver sensitivity of ~ 10^–100^ W, which is clearly impossible. Note also that based on the reported sensitivity of Yaesu VX-8 radios, and the experimentally measured 37δ operating depth depicted in Fig. 1a, the δ’/δ ratio must be no smaller than 37/15 = 2.5, which is consistent with Eq. (7) derived in the “Methods” section. This leads to an estimate of δ* > 30δ for the SEW propagation length along the water surface.

The power scaling of our radio communication experiments, which is analysed in Fig. 3 also clearly points to the SEW-mediated signal transmission. This figure compares the measured communication distances of two underwater radios operating at 30 and 50 MHz in freshwater, pool water and seawater. The 30 MHz system operates at 0.02 W power, while the 50 MHz system operates at 5 W. At both frequencies the operating distances scale linearly with the skin depth. Comparison of the radio performance at different operating powers shown in the log–log scale in Fig. 3 once again strongly favours the surface wave communication modality. Indeed, based on Eq. (1) the bulk communication channel would correspond to the much lower location of the red curve, which is indicated by the black squares and the black dashed line. Increase of transmit power by + 24 dB (from 0.02W to 5W) leads to 19 times increase of the communication distance, as compared to increase by just 2.4δ, which would be expected in the bulk communication channel. The most probable interpretation of the observed power scaling behaviour is that upon reaching the water–air interface, the radio signal propagates in a 2D fashion with the propagation distance roughly equal to 90 skin depth δ. This interpretation matches our theoretical expectations and the directly measured profile of the surface electromagnetic wave near the water surface at 30 MHz, which is shown in the inset in Fig. 3.

**Figure 3:**
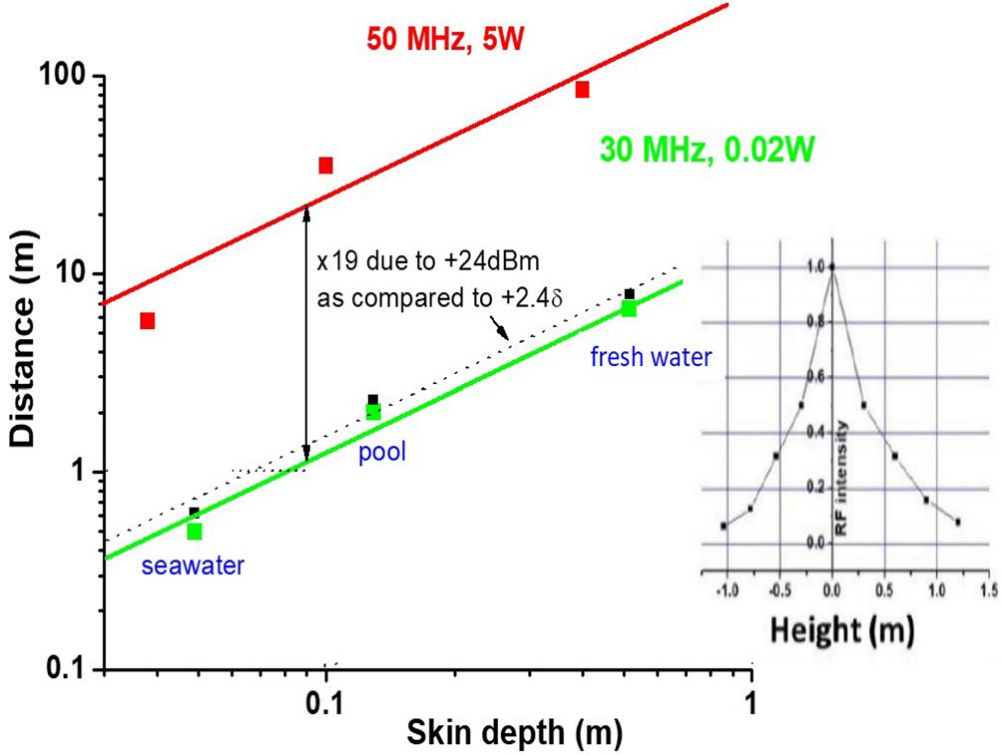
Power scaling of the underwater radio communication experiments clearly indicates the surface wave transmission mechanism. Increase of transmit power by +24 dB (from 0.02W to 5W) leads to 19 times increase of the communication distance, as compared to increase by just 2.4δ which would be expected in the bulk communication channel (indicated by the dashed line connecting black squares). Note that the measured communication distance is still proportional to the skin depth. The inset shows the measured profile of the surface electromagnetic wave near the water surface at 30 MHz, which matches theoretical expectations.

As was mentioned above, SEW-mediated radio communication through the water surface may be interpreted as a manifestation of the superlensing effect^4–6^. Even though water does not exhibit negative refractive index behavior, it supports propagation of large-wavevector SEWs^3^, which makes it possible to realize a “poor man’s superlens”^5^ (see Fig. 4a). To further confirm our understanding of this phenomenon, we have observed the superlensing effect in a dedicated laboratory experiment depicted in Fig. 4b, which was performed at 2.45 GHz. In this experiment a 20 mm layer of fresh water was used as a superlens, which was enabled by SEWs of the top water–air interface. (Note that similar to the superlensing experiments with thin metal films in the visible frequency range^5^, the superlens thickness cannot be much larger than the wavelength due to exponential attenuation of SEW field into the superlensing medium. Typically, superlens thicknesses in the λ/5–λ/10 range are used). A super-resolution image of a 10 mm diameter hole in a metal screen placed below the 20 mm layer of water was measured above the water surface by raster scanning a dipole 2.45 GHz antenna in the *xz* plane. The dipole antenna was oriented perpendicular to the water surface. Note that the free space wavelength at this frequency is λ_0_ = 125 mm, so that the ~ 20 mm wide (or ~ λ_0_/6) image produced by the water layer clearly exhibits super-resolution. These observations further confirm our understanding of radio waves propagation through water. In particular, they demonstrate the mechanism of efficient radio signal transmission through the water–air interface, which is traditionally considered to be an extremely difficult problem^8^. For a radio located above water communication looks as if it is happening with an (above surface) image of an underwater transmitter. We should note that a surface wave may also exist at the water-metal interface. However, the dielectric permittivity of a typical metal at 2.45 GHz is much larger than the dielectric permittivity of water. As a result, the SEW of the metal-water interface is much tighter localized near the interface, and its contribution to the image observed on top of the water surface should not be considerable.

**Figure 4:**
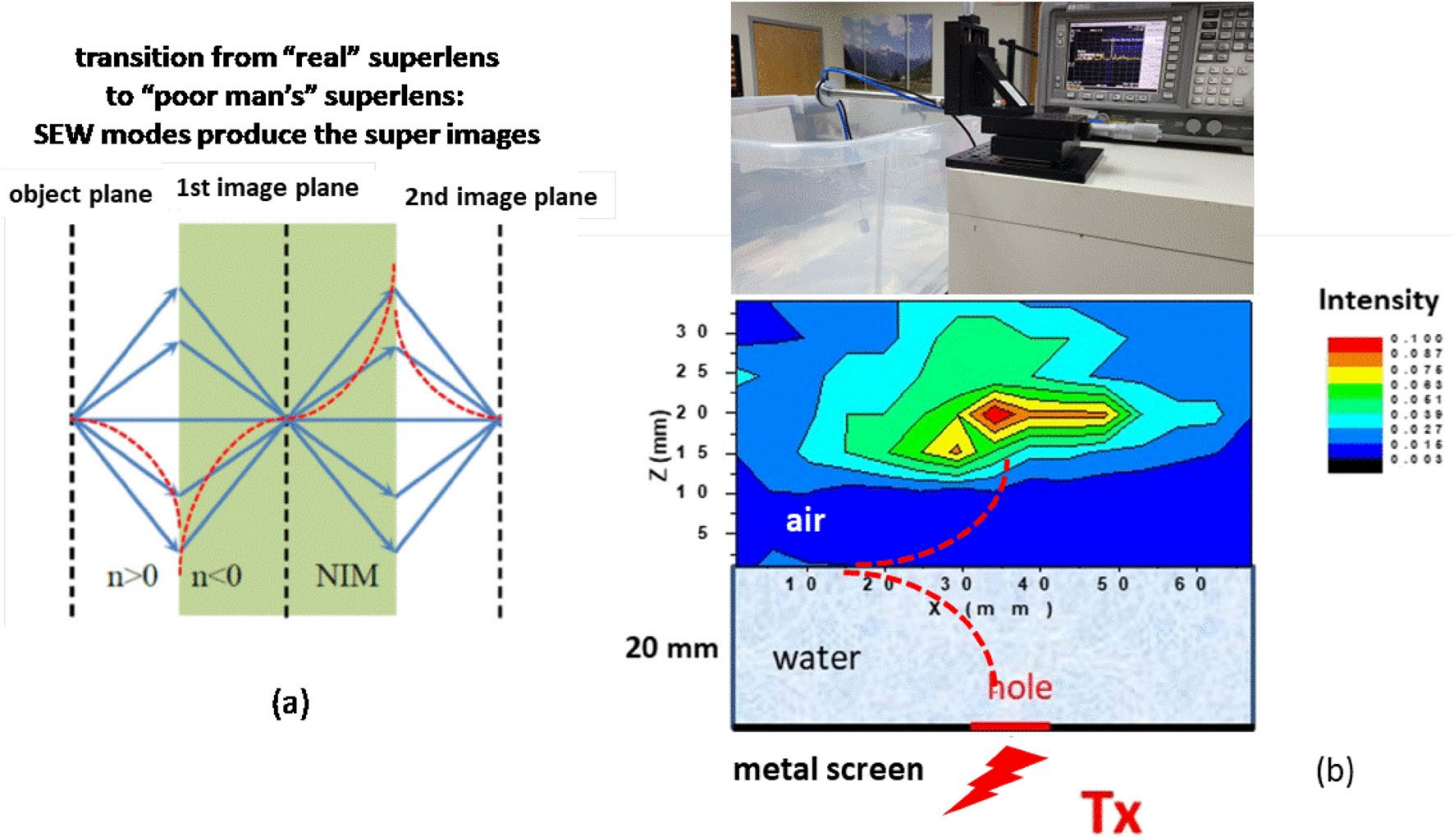
(**a**) Transition from superlens imaging using a negative refractive index material (NIM) to a “poor man’s superlens” based on a medium which supports surface electromagnetic waves (SEW). In both cases the super-resolution images are produced by the SEW components of the field. (**b**) Photo of the experimental setup and the experimentally measured 2.45 GHz image of a 10 mm diameter hole in a metal screen placed below the 20 mm layer of water. The hole is illuminated with a 2W 2.45 GHz source. The image is obtained by scanning a dipole antenna in the xz plane. In this experiment the layer of water is playing the role of a superlens. Note that the free space wavelength at this frequency is λ_0_=125 mm, so that the ~20 mm wide (or ~λ_0_/6) image produced by the water layer clearly exhibits super-resolution.

Our experiments have many important implications for fundamental and applied science. Compared to the existing state of the art in underwater radio communication, which uses extremely low frequencies^9^, we demonstrated five orders of magnitude improvement in communication bandwidth (~ 10 MHz vs. ~ 100 Hz). Since conductivity of soils (which is defined by their water content) is close to the conductivity of fresh water, our SEW-mediated techniques will open new ways to enable radio communication and imaging underground, thus addressing long standing mine safety issues. Another potentially impactful area for the described technique is oceanic and seafloor exploration. While some of these tasks are successfully performed using acoustic communication and imaging techniques, acoustic signals have much lower bandwidth and very large latency, and intense ultrasound required in these applications negatively affects marine life^10^. The use of relatively short-range low power SEW radio signals, instead of acoustics, may bring about similar results without the negative impact on marine life. Note that if increased operating distance is desired, lowering the SEW frequency down to KHz range and increasing transmit power should increase the operating distance of underwater SEW radios (see Eq. (2) and the power scaling graph in Fig. 3), and make it comparable to the operating distance of acoustic communication systems.

On the other hand, compared to the state-of-the-art visible light imaging and communication techniques used underwater^11^, SEW signals are not affected by water turbidity. We should also mention the forthcoming need for humanity to explore extraterrestrial ocean worlds, such as potentially inhabitable Jovian moons^12^. Such space missions may also benefit from the use of SEW-based underwater radios.

Our results also indicate new ways of radio waves propagation through water-containing biological tissues. The most important implication of the superlens imaging experiment depicted in Fig. 4 is that surface electromagnetic radio waves may be used in high-resolution bioimaging, in which case the human body itself may behave as a superlens for radio waves.

## Methods

### Penetration depth of surface electromagnetic waves (SEW) into water

Analytical derivation of surface electromagnetic wave solutions of the macroscopic Maxwell equations at the water–air interface may be found in Ref.^3^. If the SEW wave vector is written as

6 $$k_{x} = \frac{2\pi }{\Lambda } + \frac{i}{\delta^*}$$

(where $\Lambda$ is the SEW wavelength, and δ* is the SEW propagation length), the *k*_*z*_ component of the wave vector far from the interface may be found as

7 $$k_{z}^{2} = \varepsilon \frac{{\omega^{2} }}{{c^{2} }} - k_{x}^{2} = \left( {\varepsilon^{\prime } + i\varepsilon^{\prime \prime } } \right)\frac{{\omega^{2} }}{{c^{2} }} - \left( {\frac{2\pi }{\Lambda } + \frac{i}{\delta^*}} \right)^{2} \approx \left( {\frac{{4\pi^{2} \varepsilon^{\prime } }}{{\lambda_{0}^{2} }} - \frac{{4\pi^{2} }}{{\Lambda^{2} }} + \frac{1}{{\delta*^{2} }}} \right) + i\left( {\frac{1}{{\delta^{2} }} - \frac{4\pi }{{\Lambda \delta^*}}} \right),$$

where ε is the dielectric permittivity of water. Equation (7) demonstrates that in a broad range of SEW parameters the penetration depth δ’ of SEW into water, which is defined by the imaginary part of *k*_*z*_, may become considerably larger than the bulk skin depth δ.

### Underwater radios and SEW antennas

The underwater radios used in the experiments illustrated by Figs. 2,3 were Yaesu VX-8 radios enclosed in plastic water-tight containers. The radios were connected by water-tight coaxial cables to their respective SEW antennas described in detail in Ref.^7^. Every part of the radio system and the divers themselves were completely submerged underwater to a depth reported in the figures. The reported sensitivity of Yaesu VX-8 radio receivers at 50 MHz is 0.35 μV (or − 116 dBm). Therefore, at 5 W (+ 37 dBm) transmit power the link loss may reach up to 153 dBm, so that operation through bulk water is limited to a distance up to 15δ. On the other hand, in our experiments underwater radio communication was observed at much larger distances of several hundred skin depths, which strongly indicates the SEW-mediated mechanism of underwater radio transmission.

## Data Availability

The datasets generated during and/or analyzed during the current study are available from the corresponding author on reasonable request.
